# The interaction of genetic variants and DNA methylation of the interleukin-4 receptor gene increase the risk of asthma at age 18 years

**DOI:** 10.1186/1868-7083-5-1

**Published:** 2013-01-03

**Authors:** Nelís Soto-Ramírez, Syed Hasan Arshad, John W Holloway, Hongmei Zhang, Eric Schauberger, Susan Ewart, Veeresh Patil, Wilfried Karmaus

**Affiliations:** 1 Department of Epidemiology and Biostatistics, Arnold School of Public Health, University of South Carolina, 800 Sumter Street, Columbia, SC, 29208, USA; 2 Clinical and Experimental Sciences, Faculty of Medicine, University of Southampton, University Road, Southampton, SO17 1BJ, UK; 3 Human Development and Health, Faculty of Medicine, University of Southampton, University Road, Southampton, SO17 1BJ, UK; 4 Department of Pediatrics, Medical College of Wisconsin, 8701 W Watertown Plank Road, Milwaukee, WI, 53226, USA; 5 Department of Large Animal Clinical Sciences, Michigan State University, 3700 East Gull Lake Drive, East Lansing, MI, 48824, USA; 6 The David Hide Asthma and Allergy Research Centre, St Mary’s, Hospital, Parkhurst Road, Newport, Isle of Wight, PO30 5TG, UK

**Keywords:** Interleukin-4 receptor gene, DNA methylation, Genetic variants, Asthma, Epigenetics

## Abstract

**Background:**

The occurrence of asthma is weakly explained by known genetic variants. Epigenetic marks, DNA methylation (DNA-M) in particular, are considered to add to the explanation of asthma. However, no etiological model has yet been developed that integrates genetic variants and DNA-M. To explore a new model, we focused on one asthma candidate gene, the IL-4 receptor (*IL4R*). We hypothesized that genetic variants of *IL4R* in interaction with DNA-M at cytosine-phosphate-guanine (CpG) sites jointly alter the risk of asthma during adolescence. Blood samples were collected at age 18 years from 245 female cohort participants randomly selected for methylation analysis from a birth cohort (n = 1,456, Isle of Wight, UK). Genome-wide DNA-M was assessed using the Illumina Infinium HumanMethylation450 BeadChip.

**Results:**

Thirteen single nucleotide polymorphisms (SNPs) and twelve CpG sites of *IL4R* gene were analyzed. Based on linkage disequilibrium and association with asthma, eight SNPs and one CpG site were selected for further analyses. Of the twelve CpG sites in the *IL4R* gene, only methylation levels of cg09791102 showed an association with asthma at age 18 years (Wilcoxon test: *P* = 0.01). Log-linear models were used to estimate risk ratios (RRs) for asthma adjusting for uncorrelated SNPs within the *IL4R* gene and covariates. Testing for interaction between the eight SNPs and the methylation levels of cg09791102 on the risk for asthma at age 18 years, we identified the statistically significant interaction term of SNP rs3024685 × methylation levels of cg09791102 (*P* = 0.002; after adjusting for false discovery rate). A total of 84 participants had methylation levels ≤0.88, 112 participants between 0.89 and 0.90, and 35 between 0.91 and 0.92. For the SNP rs3024685 (‘CC’ vs. ‘TT’) at methylation levels of ≤0.85, 0.86, 0.90, 0.91, and 0.92, the RRs were 0.01, 0.04, 4.65, 14.76, 14.90, respectively (interaction effect, *P* = 0.0003).

**Conclusions:**

Adjusting for multiple testing, our results suggest that DNA-M modulates the risk of asthma related to genetic variants in the *IL4R* gene. The strong interaction of one SNP and DNA-M is encouraging and provides a novel model of how a joint effect of genetic variants and DNA-M can explain occurrence of asthma.

## Background

Asthma is a common chronic disease that affects around 235 million people around the world and 5.4 million in the United Kingdom (UK) [1]. The burden of disease affects 1.1 million children between ages 0 to 17 years in the UK. Asthma is characterized clinically by shortness of breath, wheezing episodes, chest tightness, and acute episodes of coughing [2]. The disease etiology is poorly understood and the postnatal development is not well established. Genetic susceptibility, environmental factors, and gene × environment interaction are believed to play a critical role in the development of asthma. Over 200 genes have been suggested to contribute to asthma occurrence [3-5]. The high heritability (35% to 95%) and the co-occurrence of asthma within families highlight the importance of a genetic component in disease pathogenesis [1]. In this work we focus on the interleukin receptor (*IL4R*) gene which has been clearly established as an asthma susceptibility gene in multiple candidate gene association studies [3-5].

There is evidence that interleukin-4 (IL-4) and its receptor (IL-4R) are involved in the pathogenesis of asthma [6-8]. A recent meta-analysis indicated a modest risk associated with *IL4R* single nucleotide polymorphisms (SNPs) on occurrence of asthma, but other investigators found conflicting results [7]. Analysis of asthma candidate genes in a genome-wide association study population showed that SNPs in *IL4R* were significant related to asthma with significance level between *P* = 0.05 and *P* = 0.0035 [3] despite *IL4R* not being identified in genome-wide association study (GWAS) analysis suggesting that IL4R variation is not well captured in current GWAS platforms. Other genetic regulatory mechanisms beyond DNA sequence variation may aid in explaining the role of *IL4R* in asthma. It has been suggested that epigenetic mechanisms play a role in T-cell differentiation and regulation, a crucial event in the onset of atopic diseases such as asthma [9]. Epigenetic regulatory mechanisms, such as DNA-methylation (DNA-M), may alter gene expression and protein production without changing the DNA sequence. No etiological model has yet been developed that integrates genetic variants and DNA-M. We will explore the idea that an increase of DNA-M may silence or a decrease of DNA-M may activate the effect of specific SNPs. To test this new model, we focus on one asthma candidate gene, the *IL4R* gene. We hypothesized that SNPs in interaction with cytosine-phosphate-guanine (CpG) sites jointly predispose to asthma at age 18 years. To test vertical transmission of DNA-M to offspring in future steps, this work focuses on women.

## Methods

### Study design and population

A whole-population birth cohort was established on the Isle of Wight in 1989 to prospectively study the natural history of asthma and allergic conditions. After exclusion of adoptions, perinatal deaths and refusal, 1,456 children (95%) were enrolled. The local research ethics committee approved the study and informed written parental consent was obtained for all participants at recruitment and subsequently at follow-ups, which were conducted at ages 1, 2, 4, 10, and 18 years of age. The birth cohort has been described in detail elsewhere [10,11]. In this study we focused on blood samples collected at 18 years of age from 245 female cohort participants who were randomly selected for genomic sequencing and DNA-M.

### Clinical data collection and outcome

Maternal history of asthma and smoking during pregnancy was ascertained at birth. Birth weight was obtained from birth records. At ages 1, 2, 4, 10, and 18 years, the original questionnaire-based information was updated, and weight and height of the child were measured. Breastfeeding duration was assessed at follow-up visits at ages 1 and 2 years. At age 18 years, the questionnaire-based information was updated using the International Study of Asthma and Allergies in Childhood (ISAAC) questionnaire [12]. Asthma at age 18 years was defined as subjects with a physician diagnosis of asthma plus current symptoms and/or asthma medication.

### SNP selection for the *IL4R* gene

An efficient genotype tagging scheme was developed that gave priority to variants that 1) showed strong association with asthma in the Isle of Wight birth cohort, and/or 2) have been reported by others to be associated with asthma/allergy, and/or 3) have functional importance. A literature search for *IL4R* gene plus asthma and allergy was used to identify associated variants (SNPs, indels). Functional variants included those that were non-synonymous, located in conserved DNA, and/or present in DNA regions with gene regulatory potential. Tagger implemented in Haploview 3.2 using Caucasian Hapmap data was used to develop a tagging scheme for the *IL4R* gene region, including 10 kb upstream and downstream of the gene [13]. An r^2^ value of 0.85 was the threshold for tagging and one, two and three SNP marker combination tests were used. The result was an efficient number of genotyped variants (n = 13) that would provide the needed information to statistically support or exclude the gene in its association with asthma outcomes.

### DNA methylation protocol

DNA was extracted from whole blood using a standard salting out procedure [14]. DNA concentration was determined by PicoGreen quantitation. One microgram DNA was bisulfite-treated for cytosine to thymine conversion using the EZ 96-DNA methylation kit (Zymo Research, Irvine, CA, USA), following the manufacturer’s standard protocol. Genome-wide DNA methylation was assessed using the Illumina Infinium HumanMethylation450 BeadChip (Illumina, Inc., San Diego, CA, USA), which interrogates >484,000 CpG sites associated with approximately 24,000 genes. Arrays were processed using a standard protocol as described elsewhere [15], with multiple identical control samples assigned to each bisulphite conversion batch to assess assay variability and samples randomly distributed on microarrays to control against batch effects. The BeadChips were scanned using a BeadStation, and the methylation level (beta value) calculated for each queried CpG locus using the Methylation Module of BeadStudio software.

### Exposures

The main exposures are SNPs and the methylation levels at CpG sites in the *IL4R* gene (Table 1). The following SNPs were included in the analysis: rs3024622, rs3024685, rs6498012, rs12102586, rs16976728, rs4787423, rs3024676, and rs2057768.

**Table 1:** **Location**, **position**, **and distance between the SNPs and the CpG sites in the *IL4R *gene**

		**CpG sites**											
		08932316	05729093	03980304	00090800	06641959	01706029	26937798	16649560	08317580	09791102	01165142	05903710
		TSS1500/ N_Shore	TSS1500/ Island	TSS1500/ Island	TSS200/ Island	5^′^UTR/ Island	5^′^UTR/ Island	5^′^UTR/ S_Shore	5^′^UTR	5^′^UTR	Body	Body	3^′^UTR
		**Median**, **IQR(5%,95%)**											
		0.89, 0.02 (0.86, 0.92)	0.06, 0.01 (0.04, 0.08)	0.07, 0.02 (0.04, 0.10)	0.03, 0.01 (0.01, 0.05)	0.08, 0.02 (0.05, 0.11)	0.07, 0.01 (0.05, 0.09)	0.09, 0.02 (0.06, 0.12)	0.21, 0.05 (0.15, 0.29)	0.90, 0.02 (0.87, 0.93)	0.88, 0.02 (0.85, 0.91)	0.58, 0.05 (0.51, 0.65)	0.87, 0.02 (0.84, 0.90)
**SNPs**	**Location**^#^	27324341	27324953	27325000	27325237	27325254	27325672	27326054	27338391	27345891	27353414	27367172	27375732
rs2057768													
5^′^UTR	27322095	−2246	−2858	−2905	−3142	−3159	−3577	−3959	−16296	−23796	−31319	−45077	−53637
rs6498012													
Intron	27331974	7633	7021	6974	6737	6720	6302	5920	−6417	−13917	−21440	−35198	−43758
rs3024622													
Intron	27365453	41112	40500	40453	40216	40199	39781	39399	27062	19562	12039	−1719	−10279
rs4787423													
Intron	27367334	42993	42381	42334	42097	42080	41662	41280	28943	21443	13920	162	−8398
rs3024676													
Coding	27373558	49217	48605	48558	48321	48304	47886	47504	35167	27667	20144	6386	−2174
rs3024685													
3^′^UTR	27376910	52569	51957	51910	51673	51656	51238	50856	38519	31019	23496	9738	1178
rs12102586													
3^′^UTR	27378053	53712	53100	53053	52816	52799	52381	51999	39662	32162	24639	10881	2321
rs16976728													
3^′^UTR	27381712	57371	56759	56712	56475	56458	56040	55658	43321	35821	28298	14540	5980

^#^The location is based on Build 37, also known as GRCh37. The distance was calculated by subtracting the location of the SNP to the CpG site in the *IL4R* gene. For instance, the distance between the SNP rs6498012 and the CpG site cg08932316 is 7633 (27331974–27324341).

### Statistical analysis

To assess whether our analytic sample (245 DNA samples) was representative of the total cohort available at age 18 years, we compared the characteristics of these two subsets by using the chi-square test. After cleaning the DNA-M data, beta (β) values presented as the proportion of intensity of methylated (M) over the sum of methylated (M) and unmethylated (U) sites (β = M/[c + M + U] with c being a constant to prevent dividing by zero) were used to estimate the effect of DNA methylation [16]. The methylation levels of 12 CpG sites spanning the genomic region of the *IL4R* gene (Table 1) were tested for association with asthma at age 18 years using Wilcoxon tests. Of these CpG sites, only methylation levels of cg09791102 showed a statistically significant association with asthma at age 18 years (Wilcoxon test: *P* = 0.01).

**Figure 1:**
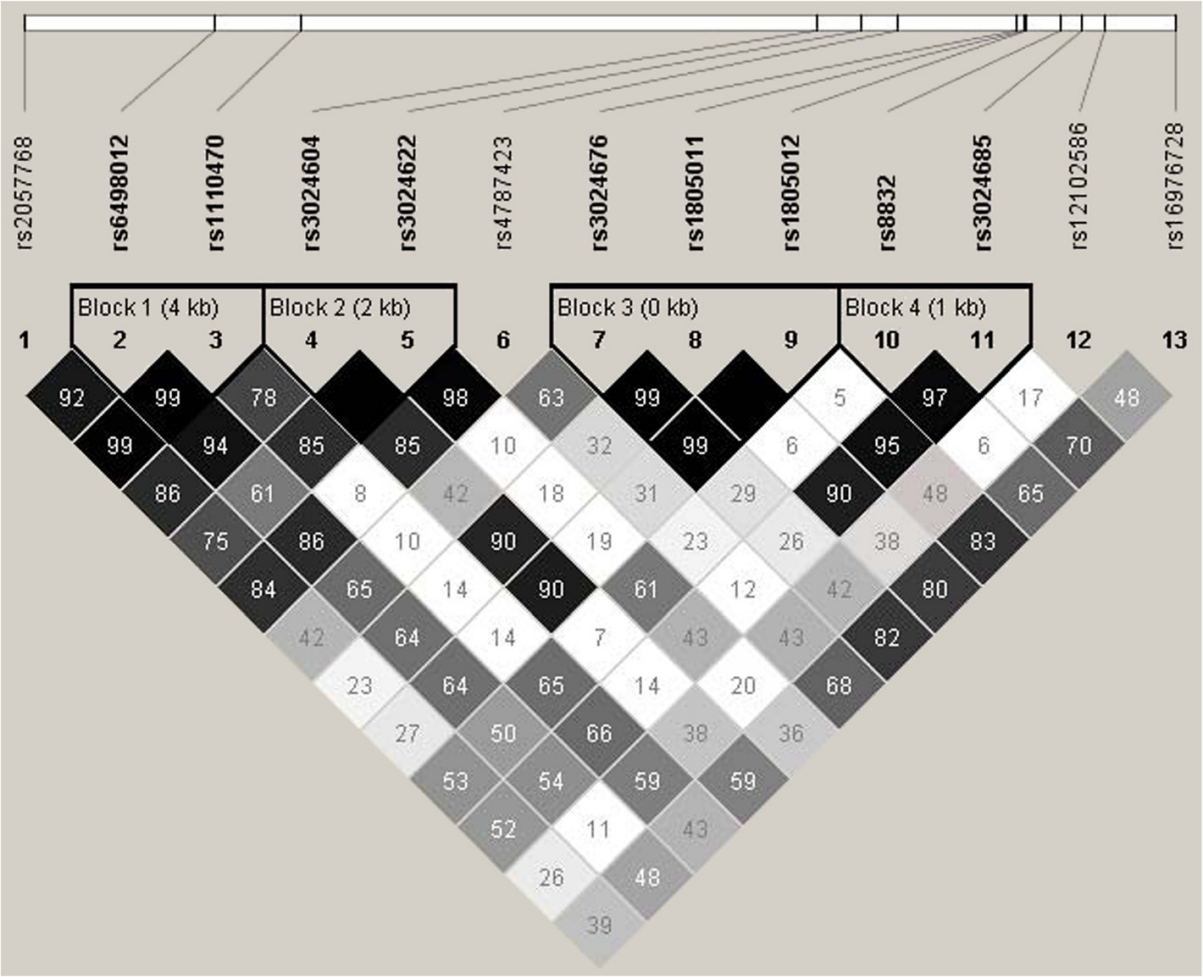
***IL4R *LD plot; standard (D’/LOD) color scheme; D’ LD values displayed.**

The 13 SNPs shown in Figure 1 were tested for Hardy-Weinberg equilibrium using Haploview 3.2 software [13] and estimates of linkage disequilibrium (LD) between SNPs were calculated using *D*’ and *r*^2^[17], to select one SNP that represents each LD block or an unlinked area.

After identifying eight uncorrelated *IL4R* SNPs (Figure 1; Table 1) and identifying which CpG site was significantly associated with asthma, we ran eight independent models to estimate statistical interactions between these SNPs and the methylation level of cg09791102 on the risk for asthma at age 18 years. We assessed the interaction on a multiplicative scale in log-linear models using an overall chi-square test as a cutoff *P* value = 0.05 for each. Only one interaction (SNP rs3024685 × cg09791102) showed a significant effect on asthma at age 18. This interaction and those SNPs and four covariates that confounded the association between the SNP and CpG interaction with asthma at age 18 years were included in the final log-linear model. We then inspected which genotype (CC, CT, or TT) explains the overall effect. Confounders include child’s BMI at age 18 (kg/m^2^), maternal history of asthma, maternal smoking during pregnancy, and breastfeeding duration (weeks). All confounders were simultaneously entered as indicator variables into the log-linear model. A backward elimination process was used to identify confounders, those that changed the association of interest by 10% or more were retained in the final model. For the reduced model, we estimated risk ratios (RR) and their 95% confidence intervals (CI).

Since we tested a total of eight crude SNP × methylation interactions before selecting the full model, we adjusted for multiple testing by applying false discovery rate (*P* = 0.05) [18]. All statistical analyses were performed using the SAS statistical package, Version 9.2 (SAS Institute, Cary, NC, USA), except for cleaning the DNA methylation data, which was done using R statistical computing package [19].

## Results

Blood samples from a subset of 245 of 750 female birth cohort participants were used to determine DNA-M at CpG sites. There were no substantial differences in prevalence of low birth weight, asthma at 18, BMI at 18, breastfeeding duration, maternal BMI, maternal history of asthma, nor maternal smoking between the female participants of the cohort and the subset included in this analysis (Table 2). For the subgroup with available methylation data 12% had maternal history of asthma, 19% had mothers that smoked during pregnancy, and 14.3% (35/245) had asthma at age 18 years.

**Table 2:** Subject characteristics with available methylation data compared to the female participants of the total cohort

	**Total female participants n(%)**	**Female with DNA methylation data**, **n(%)**	***P *value**
**Factors**	n = 750	n = 245	
**Maternal history of asthma**			
Yes	80 (10.8)	30 (12.3)	0.50
No	662 (89.2)	213 (87.7)	
Missing	8	2	
**Maternal smoking during pregnancy**			
Yes	188 (25.3)	47 (19.3)	0.05
No	555 (74.7)	197 (80.7)	
Missing	7	1	
**Maternal body mass index** (**kg**/**m**^**2**^)			
Underweight (<18.5)	10 (1.7)	4 (2.2)	0.82
Normal (18.5- <25)	355 (61.5)	109 (59.2)	
Overweight (≥25.00)	212 (36.7)	71 (38.6)	
Missing	173	61	
**Low birth weight**			
Yes	35 (4.8)	9 (3.8)	0.53
No	699 (95.2)	228 (96.2)	
Missing	16	8	
**Asthma at age 18 years**			
Yes	128 (19.4)	35 (14.3)	0.07
No	531 (80.6)	210 (85.7)	
Missing	91	0	
**Median** (**5**%, **95**% **value**); **n**			
**Breastfeeding duration** (**weeks**)	8.0 (0, 40); 664	10.5 (0, 40); 222	0.16
Missing	86	20	
**Body mass index at age 18** (**kg**/**m**^**2**^)	22.2 (18, 32); 499	22.9 (19.05, 32.93); 240	0.56
Missing	251	5	

**Figure 2:**
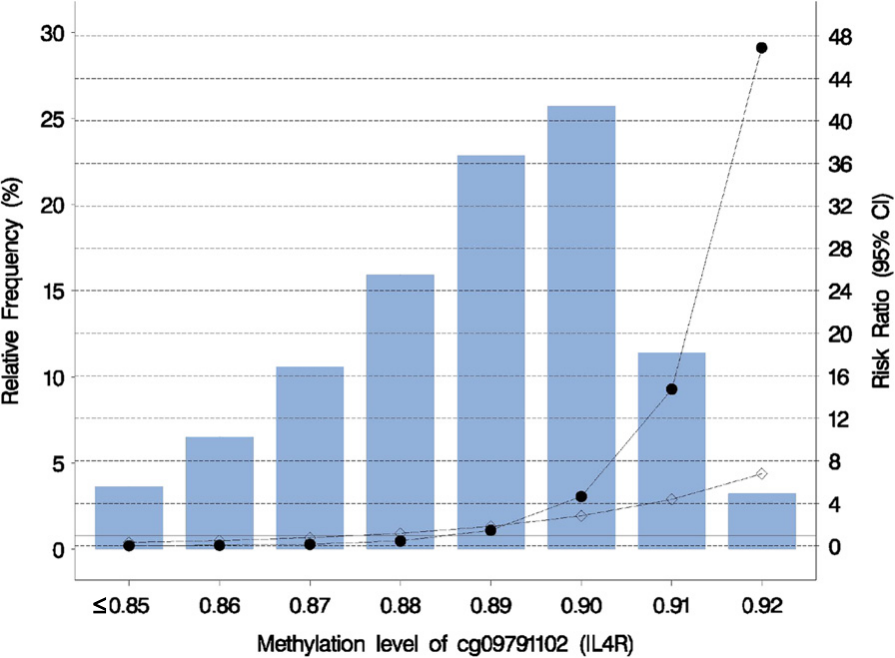
**Risk Ratio of asthma at age 18 years versus methylation score at different genotypes of *IL4R*rs3024685.** The blue bars present the relative frequency of the DNA methylation levels. For instance, 87% methylation is found in 10% of the participants. The reference genotype is ‘TT’. The solid horizontal line that indicates a risk ratio value of ‘1’ shows the risk ratio of the reference ‘TT’ genotype. The black dot represents the ‘CC’ genotype, and the diamond is ‘CT’ genotype.

Of the thirteen SNPs genotyped in the *IL4R* gene, eight SNPs were analyzed since they were uncorrelated (D’ <0.95) (Figure 1, Table 1). A total of 12 CpG sites spanning the genomic region of the *IL4R* gene were analyzed for association with asthma at age 18 years. Only methylation levels of cg09791102 showed an association with asthma at age 18 years (Wilcoxon test: *P* = 0.01). Testing for interaction between the eight SNPs and the methylation levels of cg09791102 on the risk for asthma at age 18 years, we identified that the interaction term of SNP rs3024685 × methylation levels of cg09791102 was statistically significant (*P* = 0.0003; FDR adjusted *P* value = 0.002; Table 3). In other words, the genetic risk of asthma associated with rs3024685 increases as the methylation level of cg09791102 rises (Figure 2).

**Table 3:** **Adjusted log**-**linear regression model of the interaction of genetic variants and DNA methylation of the *IL4R *gene on asthma at age 18 years**

**Parameter**		**Estimate**	**95%CI**		***P *value**
Intercept		19.59	−12.78	51.97	0.25
cg09791102		−26.97	−63.62	9.67	0.14
rs3024685	CC	−102.45	−158.51	−46.40	0.0003
	CT	−38.48	−80.71	3.75	0.07
	TT	Reference			
cg09791102* rs3024685	CC	115.54	53.18	177.91	0.0003
	CT	43.90	−3.45	91.27	0.06
	TT	Reference			
rs3024622	CC	−1.24	−3.45	0.95	0.26
	CG	−0.14	−1.00	0.72	0.74
	GG	Reference			
rs12102586	TT	2.41	0.29	4.53	0.02
	CT	0.65	−0.24	1.55	0.15
	CC	Reference			
rs16976728	TT	−0.53	−2.03	0.95	0.48
	CT	0.16	−0.78	1.11	0.72
	CC	Reference			
Maternal smoking during pregnancy		0.43	−0.40	1.26	0.31
Maternal history of asthma		0.53	−0.41	1.49	0.26
Body mass index at age 18 years (kg/m^2^)		0.05	−0.009	0.12	0.09
Breastfeeding duration (weeks)		0.02	−0.004	0.04	0.11

The DNA-M level range for cg09791102 was 0.48 to 0.92 (blue bars in Figure 2). Since the number of participants at methylation levels of 0.85 or less were low, we grouped these methylation levels into ≤0.85 (n = 9). For descriptive purposes, 84 participants had methylation levels of 0.88 and less, 112 participants of 0.89 to 0.90, and 35 of 0.91 to 0.92. Since the mode of inheritance is additive, we compared participants who had the ‘CC’ and ‘CT’ genotypes with those who were ‘TT’ genotype at rs3024685. For the genotype ‘CC’, compared to ‘TT’, we found that at methylation levels of 0.85, 0.86, 0.90, 0.91, and 0.92, the RRs of asthma were 0.01, 0.04, 4.65, 14.76, and 46.90 (Figure 2; FDR adjusted *P* value = 0.002), respectively. Similar results were found with ‘CT’ genotype, however the interaction term did not achieve statistical significance (*P* = 0.06).

Descriptively, 13.2% and 14.3% of the participants had asthma at a methylation level of 0.88 at the genotype ‘CT’ and ‘TT’, respectively; and none of the ‘CC’ genotype had asthma. Between 0.89 and 0.90 methylation levels, 15.0% of the ‘CC’, 16.7% of the ‘CT’, and 7.9% of the ‘TT’ genotype had asthma. At methylation levels larger than 0.90, 54.6% of the ‘CC’ and 16.7% of the ‘CT’ genotype had asthma, and none of the ‘TT’ genotype had asthma.

## Discussion

This is the first study to determine the role of both genetic and epigenetic factors within the genomic region of the *IL4R* gene on the risk for asthma. Although the CpG site cg09791102 is located 23,496 base pairs away from SNP rs3024685 in the intragenic region of the *IL4R* gene, we found that the risk of asthma is modulated by this CpG site even after adjusting for multiple testing. The distance between the SNP and the CpG sites is large. However, Bell *et al*. have demonstrated that for a regulation in *cis* even larger distances can show statistically significant effects [20]. Hence, these two factors (SNP and CpG site) may jointly contribute to gene expression or alternative splicing.

The SNP rs3024685 in the 3^′^UTR region has no independent effect on asthma at age 18 years; however in interaction with the CpG site cg09791102 (gene body, Table 1) it is strongly associated with asthma in female participants. At 92% methylation level, rs3024685 (‘CC’ genotype compared to ‘TT’) showed a 46.9-fold increase risk for asthma. Our observation of a role of gene-body methylation is further supported by the emerging evidence, which shows that methylation in intragenic regions can be positively correlated with gene expression levels and phenotype variation [21,22]. Intragenic DNA methylation has been linked to ‘exon definition’ through interaction with auxiliary proteins, by which DNA methylation in the body may result in alternative pre-mRNA splicing regulation (for example, inclusion or exclusion of exons) [23-25]. We assume that a higher DNA-M may mask an otherwise protective effect of rs3024685 and thus increases the risk of asthma [26]. Our results indicate that considering both genetic variants and DNA methylation will significantly improve the explanation of asthma. Replication of these findings in an independent study population is needed to validate the interplay of DNA methylation with genetic polymorphism, which results in an increased asthma risk. However, currently there are only few studies that can provide both genetic and DNA methylation data.

A limitation of our study is that the RRs at methylation levels larger than 90% are high, which is due to the limited number of individuals (n = 36) with methylation levels larger than 90%. Evidence of selection bias is absent since prevalence of asthma and *IL4R* SNPs is comparable between those analyzed in this study and those from the original cohort. Multiple testing was a concern since we tested the joint effect of differential DNA methylation of cg09791102 and eight *IL4R* SNPs separately (a total of eight tests). Nevertheless, the observed increased risk remained statistically significant after penalizing its *P* value for false discovery rate. Regarding reliability and specificity of methylation status of CpG sites, a recent report demonstrated that the Infinium HumanMethylation450 array, which was used to obtained DNA methylation profiles in this study, had strong reproducibility and high validity [27]. The extent to which DNA methylation measured in blood relate to other tissues and whether can be used as a biomarker for phenotype variation is unclear and is an area of current scientific dispute [28-30].

## Conclusions

The strong interaction of one SNP and DNA-M is encouraging and provides a novel model how a joint effect of genetic variants and DNA-M can explain asthma. Although the sample size is limited and focused on female participants, our results should generally motivate other studies to replicate the interaction we found, while also searching for new interactions between genetic variants and DNA methylation, in particular for the *IL4R* gene and asthma.

## Abbreviations

CI: Confidence interval; CpG: Cytosine-phosphate-guanine; DNA-M: DNA methylation; GWAS: Genome-wide association study; IL4R: Interleukin-4 receptor; ISAAC: International Study of Asthma and Allergies in Childhood; LD: Linkage disequilibrium; RR: Risk ratio; SNP: Single nucleotide polymorphisms; UTR: Untranslated region.

## Competing interests

The authors declare that they have no competing interests.

## Authors’ contributions

NSR conducted the statistical analysis, interpreted the data, and drafted the manuscript. SHA and VP contributed to the clinical interpretation and helped with the manuscript. JH supervised the assessment of the DNA methylation and revised the manuscript. HZ directed the statistical analysis and aided in their interpretation and the final editing. SE and ES selected and measured the single nucleotide polymorphisms. SE and SHA contributed to funding acquisition and the manuscript. WK designed the study, reviewed the data quality, helped with statistical analyses, and revised the manuscript. All authors read and approved the final manuscript.
